# Immune Regulation by Regnase‐1 and Related Endoribonucleases

**DOI:** 10.1002/eji.70313

**Published:** 2026-11-04

**Authors:** Takuya Uehata, Osamu Takeuchi

**Affiliations:** ^1^ Department of Medical Chemistry Graduate School of Medicine Kyoto University Kyoto Japan

**Keywords:** antisense oligonucleotides, mRNA decay, RNA binding proteins, stem‐loop structures

## Abstract

Regnase‐1 (ZC3H12A) is a stem‐loop (SL)‐recognizing endoribonuclease that couples immune stimulation to selective mRNA decay. Although originally characterized as a negative regulator of inflammatory cytokine expression, recent studies have expanded this view by placing Regnase‐1 within a broader network of SL‐recognizing RNA decay factors, including Roquin proteins and Regnase‐3, and by revealing context‐dependent functions in immune and nonimmune cells. Through regulation of transcripts encoding cytokines, costimulatory molecules, and transcriptional regulators, Regnase‐1 restrains innate and adaptive immune activation, but biological output varies according to cell type, tissue environment, disease stage, and mode of perturbation. In T cells, including engineered T cells used in adoptive cell therapy, Regnase‐1 inhibition can enhance antitumor activity in preclinical models, whereas in epithelial tissues Regnase‐1 can limit inflammatory circuits that affect host defense, tissue injury, and inflammation‐associated tumorigenesis. Structure‐targeting antisense oligonucleotides and genome‐editing approaches have further raised the possibility of therapeutically modulating Regnase‐dependent RNA decay. In this Review, we discuss molecular mechanisms of Regnase‐1‐mediated mRNA decay, context‐dependent functions across tissues and diseases, and the opportunities and limitations of targeting this pathway therapeutically.

## Graphical abstract

Regnase‐1 couples immune signaling to stem‐loop‐mediated mRNA decay. Its biological output depends on cellular and tissue context, determining whether it restrains inflammation, maintains tissue homeostasis, or suppresses inflammation‐associated tumorigenesis.

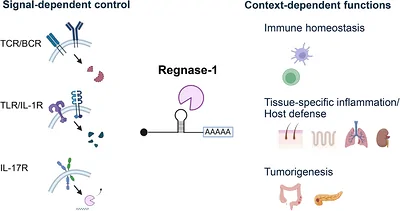

## 1 Introduction

Posttranscriptional regulation of mRNA stability represents a fundamental layer of gene expression control in the immune system, enabling rapid and adjustable tuning of inflammatory and activation‐associated transcripts [1, 2]. Among the best‐characterized RNA decay pathways are those mediated by AU‐rich element (ARE)‐binding proteins, such as tristetraprolin (ZFP36) and its paralogs ZFP36L1 and ZFP36L2. These factors recognize short AU‐rich sequences within the 3′ untranslated regions (UTRs) of target mRNAs and promote their deadenylation and decay, thereby restraining cytokine production and lymphocyte activation [3, 4, 5, 6, 7, 8, 9, 10]. Genetic studies have firmly established the importance of ARE‐mediated mRNA decay in immune homeostasis, particularly in T cells and myeloid lineages [3, 10, 11, 12, 13, 14, 15, 16, 17, 18, 19], as reviewed elsewhere [20]. In contrast to sequence‐driven ARE recognition, a distinct class of RNA decay regulators controls mRNA stability by sensing defined RNA secondary structures, most notably stem‐loop (SL) motifs in 3′ UTRs. Regnase‐1 (ZC3H12A) and the Roquin proteins (RC3H1 and RC3H2) represent this category and have emerged as key posttranscriptional gatekeepers of immune activation [21, 22] (Figure 1).

**FIGURE 1:**
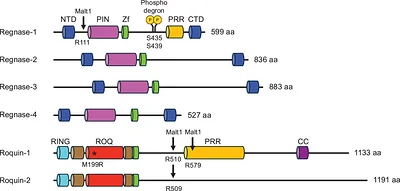
Schematic representation of the domain structures of Regnase family proteins and Roquin‐1/2. Arrows indicate MALT1 cleavage sites. Phosphorylation sites that trigger proteasome‐mediated degradation of Regnase‐1 are highlighted. The asterisk indicates the M199R mutation in the ROQ domain of Roquin‐1 (*sanroque* allele). CC, coiled‐coil domain; CTD, C‐terminal domain; NTD, N‐terminal domain; PRR, proline‐rich region; Zf, CCCH‐type zinc finger domain.

Although both Regnase‐1 and Roquin are CCCH zinc finger (Zf)‐containing RNA‐binding proteins (RBPs), they recognize target RNAs in distinct ways. Regnase‐1 harbors a PIN‐like endoribonuclease domain and a CCCH Zf that contributes to direct RNA binding and substrate positioning, thereby enabling efficient endonucleolytic cleavage of target mRNAs [23]. In contrast, RNA recognition by Roquin proteins is mediated primarily by the ROQ domain, which senses SL secondary structures, whereas the CCCH Zf plays an auxiliary role in stabilizing RNA interactions, particularly with double‐stranded regions [24, 25]. Despite recognizing structurally related SL elements, these factors engage mechanistically distinct decay pathways: Regnase‐1 directly cleaves target mRNAs as an endoribonuclease, whereas Roquin proteins primarily promote deadenylation and decapping through recruitment of the CCR4‐NOT complex and decapping‐associated machinery, including EDC4 and RCK/DDX6 [26, 27, 28].

The Regnase family comprises four paralogous members, Regnase‐1 to Regnase‐4, all of which share conserved functional domains, including a PIN‐like RNase domain and CCCH Zf motifs, suggesting a common role as RNA‐degrading enzymes (Figure 1). Among these family members, Regnase‐1 is by far the best characterized in immune regulation, whereas Regnase‐3 has recently emerged as a functionally related RNase with partially overlapping targets and cell type‐restricted functions. By contrast, the physiological roles of Regnase‐2 and Regnase‐4 in immune regulation remain much less well characterized. Several reviews have previously summarized the molecular properties of the Regnase family and Roquin proteins, as well as their roles in immune regulation [21, 22, 29, 30, 31]. Nevertheless, recent developments have further advanced our understanding of Regnase‐mediated RNA regulation. First, Regnase‐1 is increasingly understood not simply as a negative regulator of inflammatory cytokine expression, but as a signal‐responsive endoribonuclease embedded within a broader network of SL‐recognizing RNA decay factors, including Roquin proteins and Regnase‐3. In this network, overlapping RNA recognition is coupled to mechanistically distinct and context‐dependent modes of mRNA decay rather than complete functional redundancy. Second, recent studies have extended the biological scope of Regnase‐1 from immune cells to non‐hematopoietic tissues, revealing context‐dependent functions in host defense, epithelial inflammation, tissue homeostasis, and inflammation‐associated tumorigenesis. Third, Regnase‐1 has emerged as a candidate target for therapeutic modulation, including engineered lymphocyte therapies and RNA‐structure‐targeting oligonucleotide approaches. These advances highlight both the promise and the complexity of manipulating Regnase‐mediated RNA decay, as the desired biological outcome may vary depending on disease setting, target cell type, and whether the intervention is transient, tunable, or permanent.

In this review, we focus primarily on Regnase‐1, the best characterized Regnase family member in the immune regulation, while discussing Roquin proteins and Regnase‐3 where they illuminate shared SL recognition, functional overlap, and context‐dependent specialization among RNA decay pathways. We integrate recent findings on Regnase‐1 and related RNA decay factors into a conceptual framework that highlights how their biological functions are shaped by cellular and tissue context. We also highlight unresolved questions regarding target selectivity, functional overlap among SL‐recognizing RNA decay factors, and tissue specificity, as well as the opportunities and limitations of therapeutic manipulation of Regnase‐mediated RNA decay.

## 2 Functional Interplay Among SL‐Recognizing RNA Decay Factors

Regnase‐1 and Roquin are defined by their ability to recognize structured RNA elements within the 3′ UTRs of target mRNAs. CLIP sequencing analyses revealed that Regnase‐1 preferentially binds SL structures containing a pyrimidine (Y)‐purine (R)‐pyrimidine (Y) triad in the loop region [32]. In contrast, Roquin target elements were initially defined as constitutive decay elements (CDEs) within the *Tnf* mRNA (core consensus UCYRYGA) [27, 33]. Beyond canonical CDEs, Roquin recognizes a broad spectrum of U‐rich SL structures, including alternative decay elements (ADEs) with U‐rich hexaloop configurations, indicating that RNA secondary structure is the primary determinant of target recognition by the ROQ domain [24, 25, 34, 35, 36, 37, 38]. Despite their distinct historical definitions, Regnase‐1 and Roquin are now understood to recognize overlapping SL architectures, including a common class of SL structures centered on a Y–R–Y triloop configuration [32]. Consistent with this overlap, genetic ablation of either *Regnase‐1* or *Roquin* results in elevated expression of inflammatory cytokine transcripts such as *Il6* and *Tnf*, whereas combined deficiency leads to more pronounced and sustained derepression, indicating partially overlapping control of inflammatory gene expression. However, the presence of a compatible SL motif is unlikely to be sufficient to define productive regulation by a given RBP. Target selectivity may also be shaped by local sequence composition, stem and loop geometry, RNA accessibility within the 3′ UTR, and the proximity of other regulatory elements.

In line with this view, despite converging on common RNA elements, Regnase‐1 and Roquin act through mechanistically and spatially distinct pathways [32]. Regnase‐1 preferentially targets translationally active mRNAs at ribosomes in a manner dependent on the RNA helicase UPF1, enabling rapid endonucleolytic cleavage of engaged transcripts. In contrast, Roquin predominantly regulates translationally inactive mRNAs localized within processing bodies and stress granules, where it promotes deadenylation and decapping [26, 27, 28, 32]. These findings suggest that Regnase‐1 and Roquin control shared targets in a spatiotemporally segregated manner, thereby providing layered regulation of mRNA fate. Accordingly, overlap in CLIP‐defined target repertoires should not be interpreted as evidence of complete functional redundancy. Rather, CLIP analyses define shared RNA recognition potential, whereas the relative contribution of each factor to target regulation in a given physiological context may depend on cell‐type‐specific expression, subcellular localization, and translational status of the target transcript, decay mechanism, and signal‐dependent regulation of each RBP.

The principle of overlapping yet mechanistically distinct posttranscriptional control extends to adaptive immunity in vivo. In T cells, Regnase‐1 and Roquin converge on shared activation‐associated transcripts, most notably *Icos*, to constrain T‐cell activation and differentiation [39]. Genetic disruption of either pathway results in enhanced T‐cell activation, whereas combined ablation causes synergistic derepression of common target mRNAs and exacerbated immune phenotypes [39, 40]. These findings demonstrate that the overlap in target recognition is functionally relevant in vivo but also indicate that Regnase‐1 and Roquin are not simply interchangeable repressors. Rather, each factor appears to provide a separable layer of posttranscriptional control over partially shared activation‐associated transcript modules. Thus, loss of one pathway may be partially buffered by the other, whereas combined disruption removes multiple mechanistically distinct restraints on T‐cell activation. Together, these studies support a conserved regulatory logic in which Regnase‐1 and Roquin cooperate on overlapping targets while nonredundantly enforcing immune homeostasis across innate and adaptive immune compartments.

In addition to the functional overlap between Regnase‐1 and Roquin, recent work has uncovered an additional layer of cooperation within the Regnase family itself, particularly between Regnase‐1 and Regnase‐3, as demonstrated by integrative CLIP analyses revealing shared RNA targets [41]. These two RNases cooperatively regulate mRNA stability in hematopoietic stem and progenitor cells (HSPCs), thereby shaping lineage commitment during hematopoiesis [41]. In this context, the shared target Nfkbiz emerged as an important regulator of myeloid differentiation. Similarly, in mast cells, Regnase‐1 and Regnase‐3 exhibit partially redundant functions upon activation and jointly restrain the expression of inflammatory cytokine transcripts, including *Tnf*, *Il1b*, and *Il2* [42]. In contrast to Regnase‐1, Regnase‐3 is predominantly expressed in myeloid cells and has a more limited impact on inflammatory gene expression when disrupted alone [43, 44]. Notably, genetic or RNAi‐mediated loss of Regnase‐3 leads to a compensatory increase in Regnase‐1 expression, indicating that Regnase‐3 restrains Regnase‐1 levels through destabilization of the *Zc3h12a* transcript [42, 43]. These findings suggest that Regnase‐1 and Regnase‐3 can function redundantly when they are co‐expressed and engage shared inflammatory transcripts, but this redundancy is conditional rather than universal. The degree of overlap is likely influenced by lineage‐specific expression of Regnase‐3, compensatory regulation of Regnase‐1 abundance, and the activation state of the cell. In line with this context‐dependent model, Regnase‐3 also exerts a nonredundant function in specific cellular settings, such as plasmacytoid dendritic cells, where its loss results in derepression of inflammatory cytokine transcripts, including *Il6* [44].

Thus, despite overlapping SL recognition and partially shared target repertoires, Regnase‐1, Roquin proteins, and Regnase‐3 should not be viewed as interchangeable RNA decay factors. Their functional output is shaped by RNA structural features, transcript accessibility, decay mechanism, translational status, subcellular localization, cell type‐specific expression, and signal‐dependent regulation, as discussed below. Therefore, CLIP‐based overlap in RNA recognition needs to be interpreted within a broader functional framework that distinguishes shared target recognition from physiological redundancy. Moreover, CLIP‐based target identification is influenced by experimental and analytical variables, including cell type, stimulation conditions, cross‐linking methods, RNase digestion, antibody quality, sequencing depth, and computational peak‐calling strategies. Consequently, differences in CLIP‐defined target repertoires across studies may reflect technical variability as well as biological context, and physical RNA binding alone does not establish functional regulation of target mRNA stability. Such a framework can be strengthened by integrating evidence from RNA stability measurements, validation of the relevant SL element through structural analysis and mutagenesis, and matched genetic perturbation experiments in disease‐ or lineage‐relevant cellular contexts. A comparative summary of these SL‐recognizing RNA decay factors is provided in Table 1.

**TABLE 1:** Comparison of stem‐loop (SL)‐recognizing RNA decay factors in immune regulation.

Feature	Regnase‐1/ZC3H12A	Roquin‐1/2/RC3H1/RC3H2	Regnase‐3/ZC3H12C
RNA recognition	YRY triloop‐containing SL structures	CDE/ADE SL elements in 3′ UTRs, including YRY/YRN triloop‐containing CDEs and U‐rich hexaloop ADEs; recognized primarily by the ROQ domain	YRY triloop‐containing SL structures; partially overlapping SL‐containing targets with Regnase‐1
Decay mechanism	Endonucleolytic RNA decay via the PIN‐like RNase domain; UPF1‐dependent decay of translationally active mRNAs	Recruitment of CCR4–NOT complex and decapping‐associated factors, such as RCK/DDX6 and EDC4, leading to mRNA decay	Endonucleolytic RNA decay via the PIN‐like RNase domain
Cellular localization	Cytoplasm; enriched at ER/ribosome‐associated compartments	Cytoplasm; processing bodies and stress granules	Reported in macrophages to localize to phagosomal/endocytic compartments
Signal‐dependent regulation	IKK‐mediated phosphorylation followed by proteasomal degradation; MALT1‐mediated cleavage; TBK1/IKKε‐mediated phosphorylation and relocalization; 14‐3‐3‐mediated inhibition	MALT1‐mediated cleavage of Roquin‐1 and Roquin‐2	IKK‐dependent proteasomal degradation; 14‐3‐3‐mediated inhibition
Immune phenotype	Severe systemic autoimmune‐like inflammation with spontaneous lymphocyte activation, autoantibodies, and anemia upon *Regnase‐1* deficiency	M199R *sanroque* mutation causes lupus‐like disease with Tfh expansion, spontaneous GC formation, and autoantibodies; T‐cell‐specific *Roquin‐1/2* deficiency causes marked T‐cell activation and Th17‐biased inflammation	Relatively mild lymphadenopathy and systemic IFN signaling without overt autoimmunity upon *Regnase‐3* deficiency; cell‐type‐specific cytokine regulation in macrophages/cDCs and pDCs

Abbreviations: ADE, alternative decay element; cDC, conventional dendritic cell; CDE, constitutive decay element; GC, germinal center; pDC, plasmacytoid dendritic cell; RCK/DDX6, RCK/p54 DEAD‐box helicase 6; Tfh, T follicular helper; UTR, untranslated region.

## 3 Signal‐Dependent Posttranslational Control of Regnase and Roquin Proteins

Despite their distinct biochemical activities, Regnase‐1 and Roquin share several regulatory principles in lymphocytes. Both proteins contribute to T‐cell activation and differentiation, and notably, both are subject to regulated proteolysis downstream of T‐cell receptor (TCR) signaling (Figure 2). Upon TCR stimulation, the paracaspase MALT1 cleaves Regnase‐1 and Roquin [45, 46, 47, 48], thereby relieving posttranscriptional repression of activation‐associated mRNAs. In addition to these factors, the RBP N4BP1, another negative regulator of inflammatory gene expression, has also been identified as a MALT1 substrate [49]. These observations position MALT1 as an important regulatory node that coordinates transcriptional and posttranscriptional gene regulation during T‐cell activation [50, 51, 52, 53, 54, 55, 56].

**FIGURE 2:**
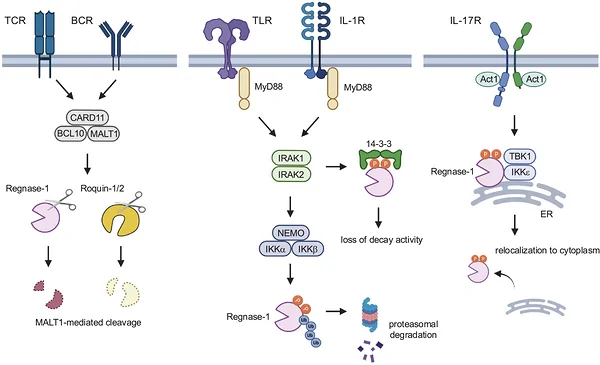
Signal‐dependent regulation of Regnase‐1 activity. Regnase‐1 is dynamically regulated by multiple immune signaling pathways. TCR signaling promotes assembly and activation of the CARD11‐BCL10‐MALT1 (CBM) complex. Activated MALT1 cleaves Regnase‐1 and Roquin proteins, which relieves posttranscriptional repression of activation‐associated mRNAs. TLR and IL‐1R signaling induces IKK‐mediated phosphorylation of the C‐terminal DSGxxS phospho‐degron (S435/S439), promoting β‐TrCP‐dependent ubiquitination and proteasomal degradation of Regnase‐1. Distinct phosphorylation events promote 14‐3‐3 binding, which functionally inhibits Regnase‐1 by suppressing target mRNA binding and mRNA decay activity. IL‐17R‐Act1 signaling induces TBK1/IKKε‐dependent phosphorylation of Regnase‐1, leading to relocalization from the ER to the cytoplasm and altered access to target mRNAs. Together, these mechanisms allow immune and inflammatory signals to dynamically tune SL‐mediated RNA decay. TCR, T‐cell receptor; TLR, Toll‐like receptor.

Regnase‐1 is further regulated by additional posttranslational mechanisms in innate and non‐hematopoietic contexts (Figure 2). Upon activation of Toll‐like receptor (TLR) and IL‐1 receptor signaling, Regnase‐1 is phosphorylated by the IKK complex at a C‐terminal DSGxxS phospho‐degron motif, triggering ubiquitin‐mediated proteasomal degradation [57]. Mice harboring S435A/S439A mutations in this degron display reduced susceptibility to experimental autoimmune encephalomyelitis (EAE), likely due to increased Regnase‐1 abundance in non‐hematopoietic compartments [58]. Moreover, IL‐17 receptor signaling induces phosphorylation of Regnase‐1 via the TBK1/IKKε complex, promoting its relocalization from the endoplasmic reticulum to the cytoplasm and dynamically modulating its access to target mRNAs [58]. Similar to Regnase‐1, Regnase‐3 is regulated by signal‐dependent proteasomal degradation in myeloid cells; however, the detailed phosphorylation and ubiquitination mechanisms remain to be elucidated [43]. In addition, phosphorylation at sites distinct from the DSGxxS phospho‐degron promotes association with 14‐3‐3 proteins, which suppresses Regnase‐1 activity; analogous 14‐3‐3–dependent inhibition has also been reported for Regnase‐3 [59, 60].

Notably, Regnase‐1 expression itself is subject to posttranscriptional control by other SL‐recognizing RNA decay factors. Both Roquin and Regnase‐3 have been shown to destabilize *Zc3h12a* mRNA, establishing cross‐regulatory circuits that fine‐tune Regnase‐1 abundance [42, 43, 47].

Collectively, these findings underscore a general principle whereby immune signaling pathways converge on posttranslational control of SL‐recognizing RNA decay factors to fine‐tune gene expression programs during immune activation.

### 3.1 Regnase‐1 in Innate Immune Cells

Regnase‐1 was originally identified as a TLR‐responsive gene, and its physiological importance became evident from genetic studies demonstrating that *Regnase‐1* deficiency results in severe systemic inflammation. *Regnase‐1*‐deficient mice exhibit early‐onset inflammatory disease characterized by growth retardation, anemia, and lymphadenopathy, reflecting a failure to restrain innate immune activation [61, 62, 63]. In macrophages, Regnase‐1 limits innate inflammatory outputs by destabilizing mRNAs encoding cytokines such as IL‐6 and IL‐12p40 (Il12b), thereby restraining excessive cytokine production upon activation of TLR signaling. A recent study implicated Regnase‐1 in alveolar macrophages in maintaining pulmonary vascular homeostasis. Conditional deletion of *Regnase‐1* in myeloid cells (using Cd11c‐cre or LysM‐cre) led to spontaneous pulmonary arterial hypertension, characterized by pulmonary arterial wall thickening and elevated right ventricular systolic pressure [64]. Transcriptomic analyses of alveolar macrophages revealed inflammatory and growth factor‐associated transcripts, including *Il6*, *Il1b*, and *Pdgfa/b*, as key Regnase‐1‐regulated mRNAs.

Recent work has shown that Regnase‐1 can restrain natural killer (NK) cell‐mediated antitumor immunity. NK cell‐specific deletion of *Regnase‐1* reduced tumor growth in vivo in a manner independent of CD8^+^ T cells [65]. *Regnase‐1*‐deficient NK cells exhibit enhanced production of IFN‐γ as well as increased expression of cytotoxic molecules, including granzymes, resulting in augmented tumor‐cell killing. Mechanistically, elevated IFN‐γ produced by *Regnase‐1*‐deficient NK cells induces CXCL16 expression in tumor‐associated myeloid cells, thereby promoting CXCR6‐dependent retention of mature NK cells within the tumor microenvironment. At the molecular level, Regnase‐1 directly targets mRNAs encoding key transcriptional regulators such as Pou2f2 (OCT2) and Nfkbiz (IκBζ), and loss of Regnase‐1 enhances OCT2‐IκBζ‐NF‐κB‐dependent transcriptional programs that drive IFN‐γ production.

### 3.2 Regnase‐1 in Adaptive Immunity

In lymphocytes, Regnase‐1 also regulates cellular activation and differentiation. This function helps maintain adaptive immune homeostasis. T‐cell‐specific deletion of *Regnase‐1* results in spontaneous T‐cell activation and autoimmune‐like inflammation, establishing a T‐cell‐intrinsic role for Regnase‐1 in restraining aberrant adaptive immune activation [46]. *Regnase‐1*‐deficient T cells display increased IFN‐γ production and clonal expansion even in the absence of overt antigenic challenge, highlighting an important role for Regnase‐1 in maintaining T‐cell quiescence. Notably, this spontaneous activation depends on TCR signaling. Restricting the TCR repertoire to an ovalbumin‐specific TCR markedly attenuates the aberrant activation of *Regnase‐1*‐deficient T cells under steady‐state conditions, whereas antigen‐dependent stimulation restores exaggerated activation and proliferation. A broader T‐cell‐intrinsic role for Regnase‐1 is further supported by independent studies showing that perturbation of Regnase‐1, either alone or together with Roquin proteins, alters T‐cell self‐reactivity and effector function, and that *ZC3H12A* loss can enhance stem‐like or persistent T‐cell states in chronic infection and engineered T‐cell settings [39, 40, 66, 67, 68]. Mechanistically, Regnase‐1 directly destabilizes mRNAs encoding key cytokines, costimulatory receptors, and transcriptional regulators involved in T‐cell activation, including IL‐2, ICOS, OX40 (Tnfrsf4), and the NF‐κB family member c‐Rel. Through this coordinated control of multiple activation‐associated transcripts, Regnase‐1 enforces tight posttranscriptional regulation of T‐cell activation and differentiation, thereby maintaining immune homeostasis. In B cells, Regnase‐1 restrains activation and differentiation programs by limiting the expression of activation‐ and survival‐associated transcripts (e.g., *Batf*, *Tnfrsf13b*, and *Bcl2*), thereby preserving germinal center (GC) homeostasis and preventing systemic immunopathology [69].

The roles of Roquin‐1 and Roquin‐2 in T‐cell activation and differentiation show striking parallels to those of Regnase‐1. A point mutation in the ROQ domain of Roquin‐1 (M199R, the *sanroque* mutation) results in severe lymphoproliferative disease characterized by increased Tfh cells, spontaneous GC formation, glomerulonephritis, and the emergence of antinuclear and anti‐dsDNA antibodies [70]. However, T‐cell‐specific deletion of *Roquin‐1* alone does not result in overt spontaneous autoimmunity. In contrast, combined ablation of *Roquin‐1* and *Roquin‐2* in T cells causes marked spontaneous activation and accumulation of effector and Tfh cells, without evidence of renal pathology [71, 72]. This phenotype is accompanied by increased levels of IL‐6 and IL‐17A in the serum and prominent Th17‐biased inflammation, particularly in the lung. Mechanistically, Roquin‐1 and Roquin‐2 regulate the expression of costimulatory molecules such as ICOS and OX40, thereby controlling Tfh differentiation. Notably, unlike *sanroque* mice or T‐cell‐specific *Regnase‐1*‐deficient mice, *Roquin‐1/2*‐deficient mice show no clear evidence of systemic autoantibody production, underscoring qualitative differences in immune pathology arising from the dominant‐negative ROQ‐domain mutation versus genetic deletion and from loss of *Regnase‐1* versus *Roquin‐1/2* among SL‐recognizing RNA decay factors.

### 3.3 Regnase‐1 in Antitumor Immunity

Given its role as a negative regulator of lymphocyte activation, Regnase‐1 has also been implicated in T‐cell‐mediated tumor immunity. Multiple independent in vivo CRISPR–Cas9 screening studies have identified Regnase‐1 as a prominent intrinsic suppressor of antitumor CD8^+^ T‐cell responses [73, 74] (Figure 3). Genetic ablation of *Regnase‐1* in OT‐I CD8^+^ T cells enhanced their accumulation, effector function, and persistence within tumors, resulting in improved tumor control across several preclinical cancer models [73, 75]. These findings have provided a rationale for applying Regnase‐1 targeting to engineered T‐cell therapies (Figure 3). Using engineered human T cells, Mai et al. showed that combined deletion of *Regnase‐1* and *Roquin‐1* further augmented antitumor cytotoxicity compared with either single perturbation alone, underscoring nonredundant posttranscriptional constraints that limit T‐cell‐mediated tumor immunity [67]. More recently, an additional layer of regulation has been revealed through the identification of the transcriptional repressor BCOR as a cooperative brake on *Regnase‐1*‐deficient T cells [76]. Dual ablation of *Regnase‐1* and *BCOR* conferred long‐term functional persistence to CAR‐T cells, highlighting how coordinated disruption of posttranscriptional and transcriptional repression can synergistically enhance the durability of antitumor T‐cell responses. In a CD19 CAR‐T model of B‐ALL, *Regnase‐1* deficiency promoted CAR‐T‐cell persistence and TCF‐1^+^ precursor exhausted T‐cell formation, leading to improved therapeutic activity in preclinical models [66]. At the molecular level, Regnase‐1 has been reported to destabilize mRNAs encoding transcriptional regulators such as BATF and TCF‐1, thereby limiting transcriptional programs that support effector function, persistence, and progenitor exhausted T‐cell formation [66, 73]. Together with the identification of BCOR as a cooperative regulator, these findings suggest that Regnase‐1‐dependent RNA decay can intersect with transcriptional and epigenetic programs that shape durable antitumor T‐cell states. However, the precise molecular pathways linking Regnase‐1‐mediated RNA decay to these transcriptional and epigenetic programs remain incompletely understood.

**FIGURE 3:**
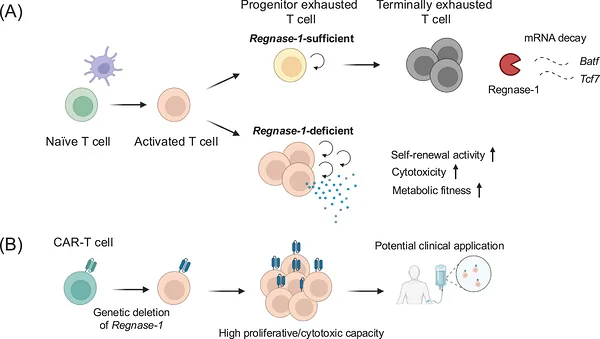
Regnase‐1 constrains CD8^+^ T‐cell fate and antitumor function. (A) Model for Regnase‐1's role in shaping CD8^+^ T‐cell differentiation in tumor microenvironment. Regnase‐1‐sufficient T cells undergo progressive differentiation toward terminally exhausted state, a process associated with Regnase‐1‐mediated destabilization of mRNAs encoding key transcriptional regulators such as BATF and TCF‐1. In contrast, *Regnase‐1*‐deficient T cells resist terminal exhaustion and instead maintain a memory‐like state characterized by enhanced self‐renewal capacity, sustained cytotoxic function, and improved metabolic fitness. (B) Schematic overview of a Regnase‐1‐targeted strategy for adoptive T‐cell therapy. In preclinical models, genetic disruption of *Regnase‐1* in CAR‐T cells enhances their proliferative capacity, persistence, and cytotoxic activity, leading to improved antitumor efficacy. This strategy may offer a potential therapeutic approach for cancer patients, although its clinical efficacy remains to be established.

Therapeutic inhibition of Regnase‐1 in T cells also raises important safety considerations. In a CD19 CAR‐T model of B‐ALL described above, *Regnase‐1* deficiency promoted CAR‐T‐cell persistence and TCF‐1^+^ precursor exhausted T‐cell formation in a largely tumor‐dependent manner [66]; in tumor‐free mice, both wild‐type and *Regnase‐1*‐deficient CAR‐T cells declined over time, and the dramatic expansion observed in tumor‐bearing mice required both CAR expression and *Regnase‐1* deletion. In contrast, combined disruption of *Regnase‐1* and *Roquin‐1* in human engineered T cells enhanced antitumor activity in solid tumor xenograft models but also increased basal inflammatory activity and caused lymphoproliferative toxicity in some mice, which was associated with extreme T‐cell expansion and could be mitigated by lowering the infused cell dose [67]. Given the enhanced activation and persistence of *Regnase‐1*‐deficient T cells, the potential risks of cytokine release syndrome (CRS) and immune effector cell‐associated neurotoxicity syndrome (ICANS) also warrant careful evaluation, although their incidence and severity in this setting remain unknown. These observations suggest that *Regnase‐1* loss may amplify antigen‐driven CAR‐T‐cell responses, but the therapeutic window can narrow when additional RNA‐regulatory brakes are removed or when in vivo expansion becomes excessive.

### 3.4 Regnase‐1 in Tissue‐Specific Inflammation and Disease

In addition to its roles in immune regulation, accumulating evidence indicates that Regnase‐1 also shapes tissue‐specific inflammatory responses, host defense, and epithelial homeostasis. Early insights came from studies of microbial infection. *Regnase‐1* haploinsufficiency confers increased resistance to *Candida albicans* infection in an IL‐17‐dependent manner [77], and mice lacking *Regnase‐1* specifically in airway epithelial cells exhibit enhanced resistance to pulmonary *Pseudomonas aeruginosa* infection [78]. Moreover, Regnase‐1 is required for effective Type 2 immune responses against helminth infection, as its dysregulation impairs ILC2 function and delayed parasite clearance [79]. These findings illustrate that the consequences of altered Regnase‐1 activity vary according to pathogen type, tissue site, and the immune or epithelial cell population involved.

The impact of Regnase‐1 on inflammatory disease is also context‐dependent. *Regnase‐1* haploinsufficiency exacerbates EAE, indicating that reduced Regnase‐1 activity can aggravate autoimmune pathology [77]. Subsequent analyses suggested that this heightened disease susceptibility can be explained, at least in part, by *Regnase‐1* haploinsufficiency in conventional Type 1 dendritic cells (cDC1s) [80]. In epithelial tissues, Regnase‐1 often acts as a cell‐intrinsic brake on IL‐17‐associated inflammation. In the skin, Regnase‐1 expression is increased in psoriatic lesions and imiquimod‐induced dermatitis [81, 82], and reduced Regnase‐1 activity exacerbates imiquimod‐induced skin inflammation by amplifying IL‐17 receptor‐driven signaling through keratinocyte–intrinsic mechanisms involving IL‐17A/IL‐17C and, in part, IL‐36 pathways [82, 83]. In the kidney, Regnase‐1 acts cell‐intrinsically in proximal tubular epithelial cells to restrain IL‐17‐driven inflammation; its loss is associated with increased *Nfkbiz* expression and exacerbated tubulointerstitial pathology [84]. Regnase‐1 also contributes to the maintenance of HSPC homeostasis by promoting the degradation of *Gata2* and *Tal1* mRNAs, thereby preventing excessive HSPC proliferation and abnormal hematopoiesis [85].

Somatic evolution in ulcerative colitis (UC) epithelium further highlights the disease‐modifying potential of the Regnase‐1‐NFKBIZ axis in IL‐17‐driven inflammation [86, 87]. Exome and targeted sequencing of inflamed UC mucosa revealed recurrent, positively selected epithelial mutations affecting IL‐17‐pathway nodes, including loss‐of‐function alterations in *NFKBIZ* and gain‐of‐function hotspot mutations in *ZC3H12A*. *ZC3H12A* mutations cluster within the DSGxxS phospho‐degron, such as S438 substitutions, where disruption of phosphorylation‐dependent ubiquitin‐mediated turnover stabilizes Regnase‐1 protein. These Regnase‐1‐enhancing mutations are not commonly observed in colorectal carcinoma tissues [86, 87]. In contrast, genetically engineered mouse models have shown that loss of *Regnase‐1* in the intestinal epithelium promotes colorectal tumor progression, largely through enhanced IL‐17 signaling driven by elevated Nfkbiz expression [88]. These findings suggest that epithelial Regnase‐1 can exert a tumor‐suppressive function in the colon by restraining inflammatory programs that fuel tumor growth. Similarly, in a spontaneous Kras^G12D^‐driven pancreatic ductal adenocarcinoma model, pancreas‐specific deletion of *Regnase‐1* accelerates tumor progression, accompanied by increased infiltration of polymorphonuclear myeloid‐derived suppressor cells (PMN‐MDSCs) and a shift toward an immunosuppressive tumor microenvironment [89].

## 4 Context‐Dependent Functions of Regnase‐1 Across Tissues and Disease Settings

The apparently divergent functions of Regnase‐1 can be reconciled by considering the cellular compartment, disease‐driving signal, target transcript module, and mode of perturbation. Regnase‐1 is often described as an anti‐inflammatory RNase because it limits the expression of cytokines, costimulatory receptors, and inflammatory transcriptional regulators. However, the biological consequence of this activity differs substantially depending on where the regulated transcripts operate. In immune effector cells, such as T cells and NK cells, Regnase‐1 restrains activation, cytokine production, persistence, and antitumor function; therefore, its disruption can enhance tumor immunity. In contrast, in epithelial tissues exposed to chronic inflammatory signals, particularly IL‐17‐associated pathways, Regnase‐1 limits inflammatory circuits such as the IL‐17‐NFKBIZ axis and thereby protects tissue integrity and may suppress inflammation‐associated tumorigenesis. This framework also helps explain observations that may otherwise appear contradictory. In chronically inflamed UC mucosa, somatic mutations that stabilize Regnase‐1 may be positively selected because they dampen excessive IL‐17‐driven epithelial inflammation. However, the mutational landscape differs markedly between non‐dysplastic UC epithelium and colitis‐associated cancer (CAC), and *ZC3H12A* mutations enriched in inflamed UC epithelium are not commonly observed in CAC. This suggests that mutations conferring a fitness advantage under chronic inflammatory stress are not necessarily maintained or may even be selected against, during neoplastic transformation. Consistent with this view, in the setting of tumor initiation or progression, epithelial Regnase‐1 can function as a tumor‐suppressive factor by restraining inflammatory programs, including the IL‐17‐NFKBIZ axis, that promote tumor growth. Defining when and how this shift in selective pressure occurs during the transition from inflamed epithelium to dysplasia and CAC will be important for evaluating the long‐term consequences of therapeutic strategies designed to enhance Regnase‐1 activity.

Another key determinant of context dependency is how Regnase‐1 activity is perturbed. Phenotypes resulting from germline deficiency, haploinsufficiency, tissue‐specific deletion, somatic mutations that stabilize Regnase‐1, transient oligonucleotide‐mediated modulation, and permanent genome editing in adoptively transferred lymphocytes should not be considered biologically equivalent. These interventions differ in cell‐type specificity, duration, reversibility, and the magnitude of Regnase‐1 modulation, all of which can shape the balance between therapeutic benefit and pathological consequence. Thus, therapeutic manipulation of Regnase‐1 should not be viewed simply as “enhancement” or “inhibition” of a single molecule, but rather as context‐specific modulation of RNA decay programs in defined cell types and disease states. Representative context‐dependent functions of Regnase‐1 across immune cells, non‐hematopoietic tissues, and disease settings are summarized in Table 2.

**TABLE 2:** Context‐dependent functions of Regnase‐1 across tissues and disease settings.

Context/Cell type	Target transcripts/modules	Consequence of Regnase‐1 perturbation	Therapeutic implication	References
Macrophages and innate immune cells	Inflammatory cytokine transcripts, including *Il6* and *Il12b*	*Regnase‐1* deficiency increases inflammatory cytokine production and causes systemic inflammatory phenotypes in genetic models	Enhancing Regnase‐1 activity may suppress selected inflammatory responses, but systemic modulation could impair protective immunity or alter tissue homeostasis	[61, 62]
Alveolar macrophages	Inflammatory and growth factor‐associated transcripts, including *Il6*, *Il1b*, and *Pdgfa*/*b*	Myeloid cell‐specific deletion causes pulmonary arterial hypertension‐like pathology in mice	Tissue‐specific effects should be considered when manipulating Regnase‐1 in inflammatory or vascular disease settings	[64]
T cells and engineered T cells	Activation‐associated transcripts, including *Il2*, *Icos*, *Ox40*, *c‐Rel*, *Batf*, and *Tcf7*	T‐cell‐specific loss causes spontaneous activation and autoimmune‐like pathology; in tumor settings, *Regnase‐1* loss can enhance antitumor T‐cell and CAR‐T‐cell function	Cell‐restricted inhibition may improve adoptive T‐cell therapies, but therapeutic window, reversibility, cytokine‐related toxicity, and antigen‐independent activation require careful control	[46, 66, 67, 73]
B cells	Activation‐ and survival‐associated transcripts, including *Batf*, *Tnfrsf13b*, and *Bcl2*	B cell‐specific loss promotes B‐cell activation and plasma cell differentiation, and autoantibody‐associated pathology	Systemic Regnase‐1 inhibition could affect humoral immunity and autoantibody‐associated pathology	[69]
NK cells	IFN‐γ‐associated programs and transcriptional regulators such as *Pou2f2* and *Nfkbiz*	NK cell‐specific deletion increases IFN‐γ production, cytotoxic molecule expression, and tumor‐cell killing in preclinical models	Regnase‐1 inhibition may enhance innate antitumor immunity, but effects on inflammatory cytokine production and tissue‐specific immune activation need evaluation	[65]
Hematopoietic stem and progenitor cells (HSPCs)	*Gata2*/*Tal1*; Nfkbiz‐mediated lineage‐bias module	*Regnase‐1* loss impairs HSPC self‐renewal; combined *Regnase‐1/3* loss promotes myeloid skewing and impaired lymphopoiesis	Systemic Regnase‐1 modulation may affect hematopoietic homeostasis and lineage balance	[41, 85]
Airway epithelium during bacterial infection	Mucosal inflammatory and immune‐regulatory pathways affecting IgA and Th17‐associated responses	Airway epithelial deletion enhances resistance to pulmonary *Pseudomonas aeruginosa* infection in mice	Inhibition may improve selected host‐defense responses, but excessive mucosal inflammation remains a potential concern	[78]
Skin keratinocytes	IL‐17/IL‐36‐associated inflammatory modules; Nfkbiz‐linked epithelial inflammatory programs	Keratinocyte‐specific loss exacerbates skin inflammation in experimental dermatitis models	Enhancing Regnase‐1 activity may be beneficial in selected epithelial inflammatory skin diseases if delivery can be restricted to the affected tissue	[81, 82, 83]
Kidney tubular epithelium	IL‐17–NFKBIZ inflammatory module	Tubular epithelial loss increases Nfkbiz expression and worsens tubulointerstitial inflammation	Tissue‐selective modulation is important because epithelial Regnase‐1 can have organ‐protective functions	[84]
Ulcerative colitis epithelium	IL‐17–NFKBIZ inflammatory module	Somatic *ZC3H12A* phospho‐degron mutations stabilize Regnase‐1 and are positively selected in chronically inflamed mucosa	Therapeutic enhancement may suppress inflammation in selected contexts, but long‐term effects on epithelial clonal dynamics and tumor susceptibility remain uncertain	[86, 87]
Intestinal epithelium in colorectal tumorigenesis	IL‐17‐NFKBIZ inflammatory tumor‐promoting programs	Epithelial deletion promotes colorectal tumor progression in mouse models	Regnase‐1 enhancement and inhibition may have different consequences depending on disease stage, oncogenic context, and duration of intervention	[88]
Pancreatic epithelium/pancreatic cancer	Inflammatory and immunosuppressive tumor microenvironment programs, including PMN‐MDSC accumulation	Pancreas‐specific deletion accelerates tumor progression and increases PMN‐MDSC infiltration in mouse models	Effects of Regnase‐1 modulation in cancer may depend strongly on epithelial context and tumor microenvironment composition	[89]

Abbreviations: CAR‐T, chimeric antigen receptor T cell; NK, natural killer; PMN‐MDSC, polymorphonuclear myeloid‐derived suppressor cell; UC, ulcerative colitis.

## 5 Artificial Nucleic Acids for the Regulation of Immune Responses

In the context of immune regulation, most current nucleic acid‐based strategies rely on antisense oligonucleotides (ASOs) that suppress mRNA expression or induce exon skipping in disease‐associated genes, as well as approaches that modulate innate immune signaling by targeting pattern‐recognition receptors such as TLRs and RIG‐I‐like receptors (RLRs) [90]. However, growing insights into RNA biology have motivated the development of next‐generation strategies that exploit endogenous posttranscriptional regulatory mechanisms rather than simply inhibiting gene expression [91].

A conceptual advance in this direction was provided by Stoecklin et al., who demonstrated that ASOs targeting SL structures recognized by Roquin can selectively stabilize *Tnf* transcripts by interfering with their regulated decay [27]. This study established that defined RNA secondary structures, rather than primary sequence alone, can be rationally targeted by artificial nucleic acids to modulate mRNA stability. Building on this principle, ASOs that disrupt Regnase‐1's recognition of SL structures have been developed to inhibit Regnase‐1‐dependent mRNA decay [32] (Figure 4). *Regnase‐1* mRNA itself contains two such SL elements within its 3′ UTR, forming an autoregulatory control of its own expression [57, 92]. ASOs designed to target these elements stabilize *Regnase‐1* mRNA, increase Regnase‐1 protein levels, and attenuate inflammatory responses in macrophages [92]. Consistent with these molecular effects, local administration showed therapeutic benefit in vivo. Intratracheal administration alleviated LPS‐induced lung injury and bleomycin‐induced pulmonary fibrosis, whereas intracranial delivery attenuated EAE, accompanied by an expansion of homeostatic microglia and regulatory T cells. Notably, Regnase‐1 expression in peripheral blood was inversely correlated with T2‐weighted MRI lesion burden in patients with multiple sclerosis, and morpholinos targeting the corresponding human SL structures suppressed inflammatory cytokine expression in human monocytic cells and primary monocytes [92]. These findings provide proof‐of‐concept that SL‐targeting oligonucleotides can modulate Regnase‐1 expression in human immune cells and support the translational potential of this approach.

**FIGURE 4:**
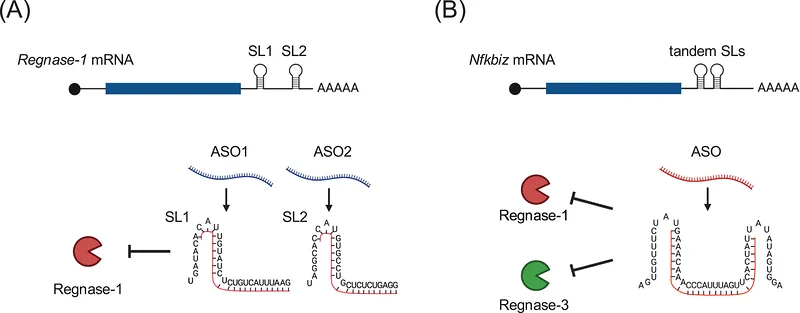
Structure‐targeting ASOs modulate Regnase‐mediated mRNA decay. (A) Schematic representation of two SL structures (SL1 and SL2) in the 3′ UTR of Regnase‐1 mRNA that mediate its autoregulation. ASO1 and ASO2 target SL1 and SL2, respectively, thereby interfering with Regnase‐1 recognition of these structures and preventing Regnase‐1‐mediated mRNA decay. (B) Schematic representation of an ASO targeting the tandem SL structures in the 3′ UTR of Nfkbiz mRNA, which are recognized by both Regnase‐1 and Regnase‐3. ASO binding interferes with recognition of these structures by Regnase‐1 and Regnase‐3, thereby preventing Regnase‐mediated mRNA decay. Arrows indicate ASO binding to the target SL structures, whereas blunt‐ended lines indicate inhibition of target RNA recognition by Regnase‐1 in (A) and by Regnase‐1 and Regnase‐3 in (B). ASO, antisense oligonucleotides; SL, stem‐loop; UTR, untranslated region.

This strategy is not restricted to *Regnase‐1* mRNA itself but can be extended to other Regnase‐regulated transcripts. Regnase‐1 and its family member Regnase‐3 have been shown to cooperatively control a subset of shared targets through SL elements [41, 42]. Among these, *Nfkbiz* mRNA emerged as a key regulator of hematopoietic stem cell fate [41]. The 3′ UTR of *Nfkbiz* harbors two tandem Regnase‐recognition SL structures separated by a short spacer, and a single ASO designed to disrupt these elements induces a pronounced bias toward myeloid differentiation in HSCs. Notably, these SL elements are also bound by Roquin, suggesting that a single ASO can simultaneously interfere with the binding of multiple SL‐recognizing RBPs to achieve robust modulation of mRNA stability. These findings raise the possibility that structure‐targeting nucleic acids may be broadly applicable to immune cells beyond HSCs, enabling cell type‐specific modulation of immune function through precise control of mRNA decay.

Nevertheless, several conceptual and translational challenges need to be considered before structure‐targeting ASOs can be broadly applied to Regnase‐mediated immune regulation. First, delivery remains a major barrier. The therapeutic effects reported to date have relied largely on local administration routes, such as intratracheal or intracranial delivery, which are suitable for selected preclinical disease models but may not be readily generalizable to systemic inflammatory diseases or to dispersed immune cell populations. Efficient delivery to the relevant tissue compartment and disease‐driving cell type will, therefore, be essential for maximizing efficacy while limiting unintended modulation of Regnase‐1‐regulated homeostatic pathways in non‐target tissues. Second, ASOs designed to disrupt SL structures may affect not only Regnase‐1 binding but also local RNA folding, neighboring regulatory elements, and the access of other SL‐recognizing RBPs, including Roquin proteins and Regnase‐3. Thus, although simultaneous interference with multiple RNA decay factors may enhance efficacy, it may also complicate mechanistic interpretation by making it difficult to attribute the observed effects solely to impaired Regnase‐1 binding. In addition, sequence‐dependent off‐target hybridization to unintended RNA transcripts represents a separate concern that must be carefully evaluated during the development of structure‐targeting ASOs. Third, from a platform perspective, the morpholino‐based oligonucleotides used in Regnase‐1‐ and Nfkbiz‐targeting studies offer a nuclease‐resistant, steric‐blocking platform for targeting RNA structures. Nevertheless, broader therapeutic application will require further optimization of oligonucleotide design and delivery strategies, including tissue distribution, cellular uptake, intracellular availability, dosing regimen, and biocompatibility in disease‐relevant settings. Finally, because Regnase‐1 functions in a tissue‐ and disease‐stage‐dependent manner, future development of Regnase‐directed ASO platforms will require careful definition of the appropriate target transcript, delivery route, duration of modulation, and therapeutic window.

## 6 Concluding Remarks

Regnase‐1 is now recognized as a signal‐responsive endoribonuclease that links immune stimulation to selective mRNA decay. By regulating transcripts encoding cytokines, costimulatory receptors, and transcriptional regulators, Regnase‐1 contributes to immune homeostasis in both innate and adaptive immune cells. Recent studies have extended this view beyond immune cells, revealing context‐dependent roles for Regnase‐1 in epithelial inflammation, host defense, tissue homeostasis, and inflammation‐associated tumorigenesis. Thus, Regnase‐1 should not be viewed simply as a universal anti‐inflammatory factor, but rather as a context‐dependent regulator whose biological output is shaped by cell type, tissue environment, disease stage, target transcript repertoire, and mode of perturbation.

Several major mechanistic questions remain unresolved. First, how Regnase target selectivity is determined remains incompletely understood. Although SL elements provide an important recognition platform, RNA structure alone is unlikely to fully explain why particular transcripts are functionally regulated in specific cellular contexts. Transcript accessibility, local sequence features, RNA‐associated regulatory elements, subcellular localization, translational state, and stimulus‐dependent regulation of RBPs are all likely to contribute. Second, the mechanisms underlying tissue specificity remain unclear. Regnase‐1 can restrain inflammatory cytokine production in immune cells, limit IL‐17‐driven epithelial inflammation, and suppress inflammation‐associated tumor growth in epithelial tissues, but the determinants that specify these divergent outcomes remain to be defined. Third, although Regnase‐1 functions within a broader network of SL‐recognizing RNA decay factors, including Roquin proteins and Regnase‐3, the physiological roles of other Regnase family members, particularly Regnase‐2 and Regnase‐4, remain poorly characterized. Defining their expression patterns, direct RNA targets, catalytic requirements, and potential redundancy or specialization relative to Regnase‐1 and Regnase‐3 will be necessary for understanding the full scope of Regnase‐mediated RNA regulation.

Another important unresolved issue is how Regnase‐mediated RNA decay is integrated with transcriptional and epigenetic programs. In antitumor T cells and engineered T‐cell therapies, Regnase‐1 has been linked to the regulation of transcriptional regulators such as BATF and TCF‐1, thereby influencing effector differentiation, progenitor exhausted T‐cell formation, persistence, and recall responses. These findings suggest that RNA decay can shape durable cellular states rather than merely tune the abundance of short‐lived inflammatory transcripts. However, how Regnase‐dependent RNA decay interfaces with chromatin accessibility, transcription factor networks, and lineage‐defining epigenetic programs remains incompletely understood.

Therapeutically, Regnase pathways offer both opportunities and risks. Genetic or functional inhibition of Regnase‐1 in engineered T cells can enhance antitumor immunity, whereas structure‐targeting oligonucleotides may provide a tunable means of modulating Regnase‐dependent RNA decay in inflammatory diseases. However, these strategies must be balanced against the homeostatic role of Regnase‐1 in preventing excessive immune activation. An important challenge will be to determine whether Regnase pathways can be manipulated with sufficient cell‐type specificity, appropriate duration, and controlled magnitude to achieve therapeutic benefit without inducing systemic autoimmunity, cytokine‐related toxicity, impaired protective immunity, or unintended tissue‐specific effects. Therefore, the development of Regnase‐directed therapeutic strategies will require not only effective molecular interventions, but also careful definition of disease context, target cell population, delivery strategy, reversibility, and therapeutic window. Addressing these challenges should help clarify how signal‐responsive RNA decay programs can be selectively rewired to modulate immune and inflammatory disease states.

## Author Contributions

Takuya Uehata and Osamu Takeuchi wrote the manuscript and prepared the figures/tables. Both authors read and approved the final manuscript.

## Conflicts of Interest

The authors declare no conflicts of interest.

## Data Availability

Data sharing is not applicable to this article as no datasets were generated or analyzed during the current study.
